# Altered Mitochondrial Respiration and Other Features of Mitochondrial Function in *Parkin*-Mutant Fibroblasts from Parkinson's Disease Patients

**DOI:** 10.1155/2016/1819209

**Published:** 2016-03-08

**Authors:** William Haylett, Chrisna Swart, Francois van der Westhuizen, Hayley van Dyk, Lize van der Merwe, Celia van der Merwe, Ben Loos, Jonathan Carr, Craig Kinnear, Soraya Bardien

**Affiliations:** ^1^Division of Molecular Biology and Human Genetics, Faculty of Medicine and Health Sciences, Stellenbosch University, Cape Town, South Africa; ^2^Department of Physiological Sciences, Faculty of Science, Stellenbosch University, Stellenbosch, South Africa; ^3^Centre for Human Metabolomics, Faculty of Natural Sciences, North-West University, Potchefstroom, South Africa; ^4^Department of Statistics, University of the Western Cape, Cape Town, South Africa; ^5^Division of Neurology, Faculty of Medicine and Health Sciences, Stellenbosch University, Cape Town, South Africa; ^6^SA MRC Centre for Tuberculosis Research and the DST/NRF Centre of Excellence for Biomedical Tuberculosis Research, Stellenbosch University, Cape Town, South Africa

## Abstract

Mutations in the* parkin* gene are the most common cause of early-onset Parkinson's disease (PD). Parkin, an E3 ubiquitin ligase, is involved in respiratory chain function, mitophagy, and mitochondrial dynamics. Human cellular models with* parkin* null mutations are particularly valuable for investigating the mitochondrial functions of parkin. However, published results reporting on patient-derived* parkin*-mutant fibroblasts have been inconsistent. This study aimed to functionally compare* parkin*-mutant fibroblasts from PD patients with wild-type control fibroblasts using a variety of assays to gain a better understanding of the role of mitochondrial dysfunction in PD. To this end, dermal fibroblasts were obtained from three PD patients with homozygous whole exon deletions in* parkin* and three unaffected controls. Assays of mitochondrial respiration, mitochondrial network integrity, mitochondrial membrane potential, and cell growth were performed as informative markers of mitochondrial function. Surprisingly, it was found that mitochondrial respiratory rates were markedly higher in the* parkin*-mutant fibroblasts compared to control fibroblasts (*p *= 0.0093), while exhibiting more fragmented mitochondrial networks (*p* = 0.0304). Moreover, cell growth of the* parkin*-mutant fibroblasts was significantly higher than that of controls (*p* = 0.0001). These unanticipated findings are suggestive of a compensatory mechanism to preserve mitochondrial function and quality control in the absence of parkin in fibroblasts, which warrants further investigation.

## 1. Introduction

Parkinson's disease (PD) is a progressive and debilitating neurodegenerative disorder, characterized by a distinct motor phenotype and the selective loss of dopaminergic neurons in the substantia nigra. While the etiology of PD is not fully understood, it is thought to involve a combination of different genetic, cellular, and environmental factors that independently or concurrently contribute to neurodegeneration. To date, several PD-causing genes have been identified, and investigations of their function have provided novel insights into the pathobiology of this disease [1].

Recently, particular attention has been drawn to* parkin*, mutations in which are the most common genetic cause of early-onset PD. Over 200 different pathogenic* parkin *mutations have been reported to date, including missense and nonsense mutations, small insertions/deletions (indels), and large whole exon deletions and duplications, across various ethnic groups [2, 3]. Parkin is a RING-between-RING- (RBR-) type E3 ligase that ubiquitinates protein substrates and targets such substrates for degradation via the ubiquitin proteasome system (UPS). Therefore, the loss of functional parkin may result in the deleterious dysregulation of its substrates and may lead to cellular dysfunction and neuronal cell death [4].

Parkin's enzymatic activity has also been implicated in the maintenance of mitochondrial health, and mitochondrial dysfunction is commonly reported in animal models of parkin deficiency [5]. For example,* parkin*-knockout* Drosophila* demonstrate prominent mitochondrial abnormalities, muscle degeneration, and dopaminergic degeneration [6–8]. While* parkin*-knockout mice exhibit milder neurological deficits, they consistently show mitochondrial impairment and oxidative damage [9, 10]. Recent studies have elegantly demonstrated the pivotal role of parkin in promoting the sequestration and autophagic degradation of damaged mitochondria (mitophagy) [11–14]. Upon mitochondrial depolarization, parkin is recruited to the outer mitochondrial membrane (OMM) through direct phosphorylation by PINK1, where it ubiquitinates several OMM proteins [15–18]. This widespread ubiquitination of OMM proteins results in the recruitment of the autophagy machinery and the autophagic clearance of damaged mitochondria, promoting cell survival [19].

In addition to its important role in mitophagy, parkin is also involved in the regulation of mitochondrial fission and fusion, continuous processes that orchestrate a dynamic cellular network of mitochondria. These processes fine-tune the mitochondrial network in response to changes in the metabolic environment, in order to maintain favorable mitochondrial function during metabolic perturbations [20]. Moreover, parkin promotes mitochondrial biogenesis via its UPS-dependent regulation of the PARIS-PGC-1*α* pathway [21]. Interestingly, parkin also directs the localized translation of mitochondrial respiratory chain component mRNA at the OMM [22]. Hence, it is evident that parkin plays important roles in the promotion and coordination of various aspects of mitochondrial health, including degradation of damaged mitochondria, mitochondrial dynamics, and mitochondrial biogenesis. It is hypothesized that dysregulation of the careful balance between these processes may significantly compromise mitochondrial health [23]. However, the exact role of mitochondrial function in the pathogenesis of PD remains largely unclear.

Notably valuable in the investigation of PD-associated mitochondrial dysfunction are patient-derived primary cell models of PD [24].* Parkin*-mutant dermal fibroblasts in particular are a useful and easily accessible tool to study mitochondrial phenotypes in an* ex vivo* setting. However, previous studies of fibroblasts from patients with* parkin* mutations have been inconsistent [25–29]. We have previously reported subtle mitochondrial abnormalities in dermal fibroblasts obtained from three South African early-onset PD patients carrying homozygous loss-of-function* parkin* mutations [28]. The present study serves to follow-up our previous report with a more comprehensive analysis of mitochondrial respiration, and with the inclusion of three age- and gender-matched control individuals.

## 2. Materials and Methods

### 2.1. Study Participants and Tissue Culture

This study gained ethical approval from the Health Research Ethics Committee of Stellenbosch University, Cape Town, South Africa (Protocol number 2002/C059). Written informed consent was obtained from all participants.

Dermal fibroblasts were previously obtained from three South African PD patients with homozygous* parkin* mutations, namely, patient 1 (P1) and a pair of affected siblings patients 2 and 3 (P2 and P3) [28]. All three patients underwent a standardized examination by a movement disorder specialist (JC) and met the UK Parkinson's Disease Society Brain Bank diagnostic criteria for PD diagnosis [30]. P1 presented with mild dyskinesia, resting tremor, and dystonia of the left leg and responded well to levodopa therapy. Both P2 and sibling P3 presented with typical PD features as well as dystonia, while P3 exhibited greater disease severity. Each patient's mutation status (P1, homozygous* parkin* exon 3-4 deletion; P2 and P3, homozygous* parkin* exon 4 deletion) was confirmed by means of multiplex ligation-dependent probe amplification (MLPA) analysis and cDNA sequencing, as previously reported [31, 32].

Three age- and gender-matched control individuals were also used, namely, Ct1, Ct2, and Ct3. The three controls had no history of neurological disease and were confirmed to be wild-type with regard to the* parkin* gene by means of cDNA sequencing. Relevant genotypic and phenotypic details of the three PD patients and three controls are summarized in Table 1.

Dermal fibroblasts were obtained from P1, P2, P3, and Ct1 by means of skin punch biopsies taken from the inner upper arm. Ct2 and Ct3 fibroblast cell lines were purchased from Sciencell Laboratories (USA) and were selected to be age- and gender-matched to patient fibroblasts. Fibroblasts were cultured in Dulbecco's Modified Eagle's Medium (DMEM, Lonza, Switzerland) with 4.5 g/L glucose supplemented with 10% (v/v) fetal bovine serum, 1% (v/v) L-glutamine, and 1% (v/v) penicillin/streptomycin, in a 5% CO_2_ humidified incubator at 37°C. All experiments were performed on fibroblasts with comparable passage numbers, ranging from 6 to 12, in order to avoid possible effects of cellular senescence.

### 2.2. Mitochondrial Respiration Analysis

Measurements of mitochondrial respiration are strong indicators of the functional bioenergetic capacity of mitochondria, and of overall cellular health. The Seahorse Extracellular Flux Analyzer uses a plate-based approach and fluorescence detectors to accurately and simultaneously measure cellular oxygen consumption rates (OCR) of multiple samples in real time [33]. Moreover, the Seahorse Analyzer allows for the sequential addition of pharmacological reagents to probe the function of individual components of the mitochondrial respiratory chain in a single experiment. This can be expressed as various informative parameters of mitochondrial function [34].

Mitochondrial respiration assays were performed using a Seahorse XF96 Cell Mito Stress Test Kit (Seahorse Biosciences, USA), in accordance with manufacturer's instructions. Briefly, fibroblasts were seeded at an optimized density of 22 000 cells per well in a 96-well Seahorse cell culture plate and incubated overnight. Each fibroblasts cell line was seeded in eight replicate wells (*N* = 8). After 24 h, the Seahorse XF^e^96 Extracellular Flux Analyzer (Seahorse Biosciences, USA) along with XF^e^ Wave software (Seahorse Biosciences, USA) was used to measure the OCR of each well. A period of 1 h before the measurements was initiated, the culture media in each well was replaced with 175 *μ*L of Seahorse assay media supplemented with 1 mM pyruvate, and the plate was further incubated for 1 h at 37°C without CO_2_. Thereafter, successive OCR measurements was performed for each well, consisting of three basal OCR measurements, three OCR measurements following the automated injection of 1 *μ*M oligomycin, three OCR measurements following the injection of 1 *μ*M carbonyl cyanide p-trifluoromethoxyphenyl hydrazone (FCCP), and finally three OCR measurements following the dual injection of 1 *μ*M rotenone and 1 *μ*M antimycin A. Subsequently, relative DNA content in each well of the plate was measured using a CyQUANT® cell proliferation assay kit (Life Technologies, USA).

The Seahorse assays were analyzed using XF^e^ Wave software, according to manufacturer's instructions. All OCR measurements were normalized to cell number and used to calculate various mitochondrial parameters, including basal mitochondrial OCR, OCR due to the proton leak across the inner mitochondrial membrane, OCR due to ATP synthesis, ATP coupling efficiency, and maximum OCR and spare respiratory capacity, following established methods [34]. The minimum OCR after rotenone and antimycin A injection was interpreted as the OCR due to nonmitochondrial respiration, and this rate was subtracted from all other measurements in order to isolate mitochondrial OCR.

### 2.3. Mitochondrial Network Analysis

Mitochondrial morphology of fibroblast cells was assessed by means of live-cell fluorescence microscopy, where staining with the Mitotracker® Red dye was used to visualize the mitochondrial network. Cells were seeded at a density of 3000 cells per well in a Nunc® Lab-Tek® 8-well chamber slide (Thermo Scientific, USA), which was then incubated overnight. Subsequently, cells were stained with a 100 nM solution of Mitotracker Red CMXRos (Life Technologies, USA) and imaged inside a live-cell environmental chamber of an Olympus IX-81 motorized inverted fluorescence microscope (Olympus Biosystems GmbH, Japan) equipped with a F-view-II cooled CCD camera (Soft Imaging Systems, Germany). Fluorescence was excited through a 572 nm excitation filter, and fluorescence emission collected at 599 nm using a UBG triple-bypass emission filter cube and an Olympus Plan AP N 60X/1.42 oil-immersion objective. All images were acquired as Z-stacks, with 7–12 image frames per stack and increments of 0.26–0.3 *μ*m between frames, using Cell^∧^R imaging software (Olympus Biosystems GmbH, Tokyo, Japan).

Following image acquisition, micrographs were deconvoluted in order to remove out-of-focus fluorescent signal. Cells were individually analyzed using ImageJ Software version 1.47 (http://imagej.nih.gov/ij/) with an average of 40 cells analyzed per sample. Raw images were binarized and optimized by manual contrast adjustment. The individual morphological characteristics of the mitochondria within a given cell, such as area, perimeter, and major and minor axes, were measured and used to calculate aspect ratio (ratio between the major and minor axes of the ellipse equivalent to the mitochondrion) and form factor (perimeter^2^/(4*π* × area)) [25]. Aspect ratio is consistent with mitochondrial length, whereas form factor is a quantification of the degree of branching of the mitochondrial network.

It should be noted that while the mitochondrial respiratory and network analyses was performed on all three patient-derived fibroblast cell lines P1, P2, and P3, due to microbial contamination of the stocks of P1's fibroblasts these cells had to be discarded; therefore only P2 and P3 were available for the assays of Δ*ψ*_*m*_ and cell growth.

### 2.4. Mitochondrial Membrane Potential Analysis

In the present study, mitochondrial membrane potential (Δ*ψ*_*m*_) was assessed with the tetraethyl benzimidazolyl carbocyanine iodide (JC-1) cationic dye and flow cytometric analysis. JC-1 exhibits potential-dependent accumulation in mitochondria, resulting in a fluorescence emission shift from 525 nm (green) to 590 nm (red). Therefore, loss of Δ*ψ*_*m*_ is detectable by the decrease in the red : green fluorescence emission ratio [35].

Cultured fibroblasts were incubated with 0.5 *μ*g/mL JC-1 (Life Technologies, USA) in the dark for 1 h. The stained cells were collected, rinsed, and resuspended in prewarmed sterile phosphate-buffered saline (PBS). JC-1 dye equilibration was allowed for 10 min at room temperature, after which the stained cell suspensions were immediately analyzed on a BD FACSCalibur flow cytometer (Becton Dickinson, USA) using BD CellQuest PRO software (Becton Dickinson, USA). The JC-1 fluorophore was excited with a 488 nm argon-ion laser after which red and green emission were separately detected in the FL1 and FL2 channels, respectively, using standard PMT detectors. Debris and aggregates were gated out by establishing a population of interest based on forward scatter/side scatter (FSC/SSC) properties. Compensation between FL1 and FL2 was carefully adjusted in reference to a CCCP-treated positive control sample, according to the manufacturer's instructions. A total of 10 000 events were collected per sample in each of three separate experiments (*N* = 3).

### 2.5. Cell Growth Assays

Cell growth rate is considered to be one of the most sensitive and reliable indicators of overall cellular health [36]. The present study investigated cell growth of fibroblasts by means of CyQUANT assays, which measures cellular DNA content via fluorescent dye binding. As DNA content is tightly regulated, CyQUANT assays can be used as accurate measurements of cell number. Fibroblasts were seeded in quadruplicate into a 96-well plate at a density of 5000 cells per well and left to adhere overnight. Culture media was then replaced and supplemented with either 10 *μ*M carbonyl cyanide m-chlorophenylhydrazone (CCCP, to induce cellular stress) or 0.1% (v/v) ethanol (vehicle control), and the plate incubated further for 24 h. Cell growth assays were performed using a CyQUANT NF Cell Proliferation Kit (Life Technologies, USA), according to manufacturer's instructions. Briefly, adherent cells in the 96-well plate were gently rinsed once with prewarmed sterile PBS and a volume of 100 *μ*L of 1x dye binding solution was added to each well. The plate was then incubated in the dark for 1 h. Subsequent fluorescence intensity was measured in a Synergy HT luminometer (BioTek, USA) with excitation at 480 nm and emission detection at 530 nm.

### 2.6. Statistical Analysis

Linear mixed-effects modeling was used to compare grouped patient-derived and control fibroblasts, with groups as fixed effects. The freely available program R, a language and environment for graphics and statistical computing (https://www.r-project.org/), and R packages* nlme* and* effects* were used [37]. Adjustments were made for the effect that the different observations on a specific cell line will be correlated. Separate experimental runs were modeled as random effects. Where appropriate, a 2^2^ factorial design was used to model effects of pharmacological treatment on outcomes. Outcome distributions were graphically depicted as boxplots with indicated medians. Where appropriate, notched boxplots were used to indicate the 95% confidence intervals of the medians. For analysis of mitochondrial network morphology, all outcome distributions were transformed (taking the natural logarithm) in order to approach normality, as the untransformed distributions of form factor and aspect ratio were positively skewed. Results were not adjusted for multiple testing because it has been suggested that corrections, such as Bonferroni, are too conservative when several associations are tested in the same group of individuals [38]. All *p* values were derived from the results of the specific models, where *p* values of < 0.05 were considered to be of nominal statistical significance.

## 3. Results

### 3.1. Mitochondrial Respiratory Rates Are Elevated in* Parkin*-Mutant Fibroblasts

**Figure 1:**
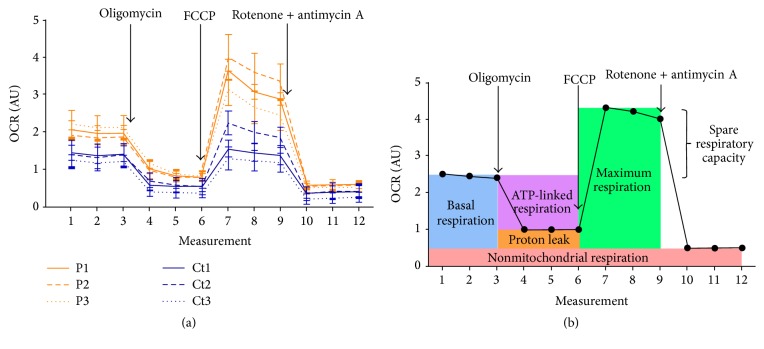
Respiratory flux profiles of patient-derived and control fibroblasts, as determined by a Seahorse Extracellular Flux Analyzer with twelve consecutive measurements of oxygen consumption rate (OCR). Addition of ATP synthase inhibitor oligomycin, electron transport chain uncoupler FCCP and complex I and III inhibitors rotenone and antimycin A are indicated. (a) Respiratory flux profiles of patient-derived and control fibroblasts. Results are expressed as mean ± SEM. (b) Illutrative respiratory flux profile indicating various parameters of respiratory control. These include: OCR due to non-mitochondrial respiration (rotenone/antimycin A response); basal mitochondrial OCR (basal measurement minus rotenone/antimycin A response); ATP-linked OCR (basal measurement minus oligomycin response); OCR due to proton leak (oligomycin response minus rotenone/antimycin A response); ATP coupling efficiency (basal mitochondrial OCR divided by ATP-linked OCR); maximum OCR (FCCP response minus rotenone/antimycin A response) and spare respiratory capacity (maximum OCR divided by basal mitochondrial OCR). AU, arbitrary units; Ct, control; OCR, oxygen consumption rate; P, patient; SEM, standard error of the mean.

In order to compare the bioenergetic status of the three* parkin*-mutant and three wild-type control fibroblasts, mitochondrial respiration analyses were performed. All OCR readings were normalized to cell number. The overall respiratory responses of all patient-derived and control fibroblasts are illustrated in Figure 1(a), from which several important respiratory parameters can be assessed (Figure 1(b)).

**Figure 2:**
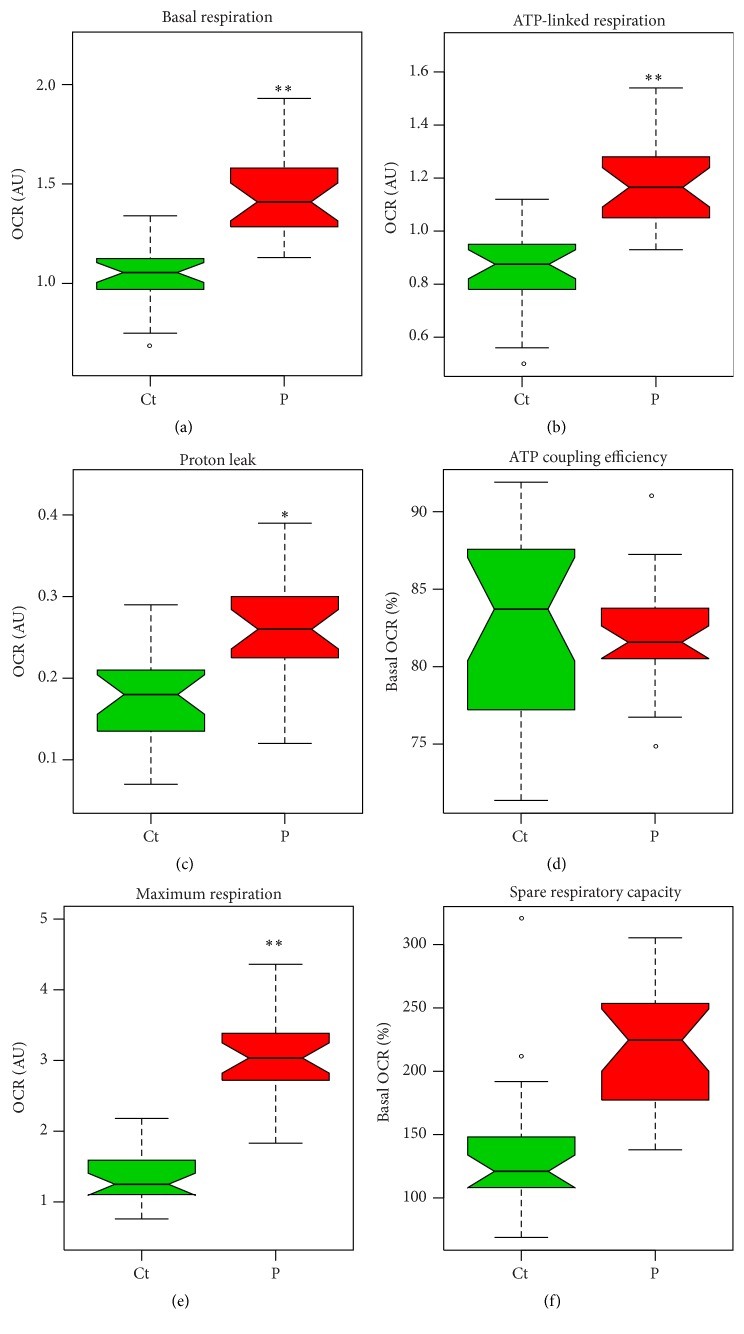
Parameters of respiratory control in patient-derived and control fibroblasts. Boxplots depict grouped patients (P) and control (Ct) values. (a) Basal mitochondrial OCR. (b) ATP-linked OCR. (c) OCR due to proton leak. (d) ATP coupling efficiency (percentage OCR due to ATP synthesis). (e) Maximum OCR. (f) Percentage spare respiratory capacity. ^*∗*^ *p* < 0.05; ^*∗∗*^ *p* < 0.01; °, outlier; AU, arbitrary units; OCR, oxygen consumption rate.

A comparison of these parameters in grouped* parkin*-mutant and wild-type control fibroblasts is provided in Figure 2. Patient-derived fibroblasts had a markedly higher mitochondrial respiration than control fibroblasts under basal conditions (*p* = 0.0093; Figure 2(a)). This mitochondrial respiration is composed of two components: the oxygen consumption devoted to ATP synthesis and the oxygen consumption due to the natural proton leak across the inner mitochondrial membrane. The addition of the ATP synthase inhibitor oligomycin allowed for these contributory components to be isolated. While* parkin*-mutant fibroblasts demonstrated higher proton leak (*p* = 0.0375; Figure 2(b)), the elevation in ATP-linked respiration was more pronounced in these cells (*p* = 0.0060; Figure 2(c)). A comparison of the ATP-coupling efficiency demonstrated similar coupling efficiencies in* parkin*-mutant and control cells (*p* = 0.5541; Figure 2(d)), suggesting that the lack of parkin did not significantly impair respiratory efficiency in the patient fibroblasts.

The addition of the accelerator FCCP allowed for an estimation of the maximum, uncontrolled OCR. FCCP is an ionophore which directly transports protons across the inner mitochondrial membrane instead of via the ATP synthase proton channel. Hence, addition of FCCP collapses Δ*ψ*_*m*_, leading to a rapid consumption of oxygen without the generation of ATP. The maximal respiratory rate is determined by several factors, including the functional capacity of the electron transport chain. It was found that the patient-derived cells had a markedly higher maximum respiratory rate than control fibroblasts (*p* = 0.0081; Figure 2(e)), whereas spare respiratory capacity was comparable between these two groups (*p* = 0.1145; Figure 2(f)).

### 3.2. *Parkin*-Mutant Fibroblasts Demonstrate More Fragmented Mitochondrial Networks

**Figure 3:**
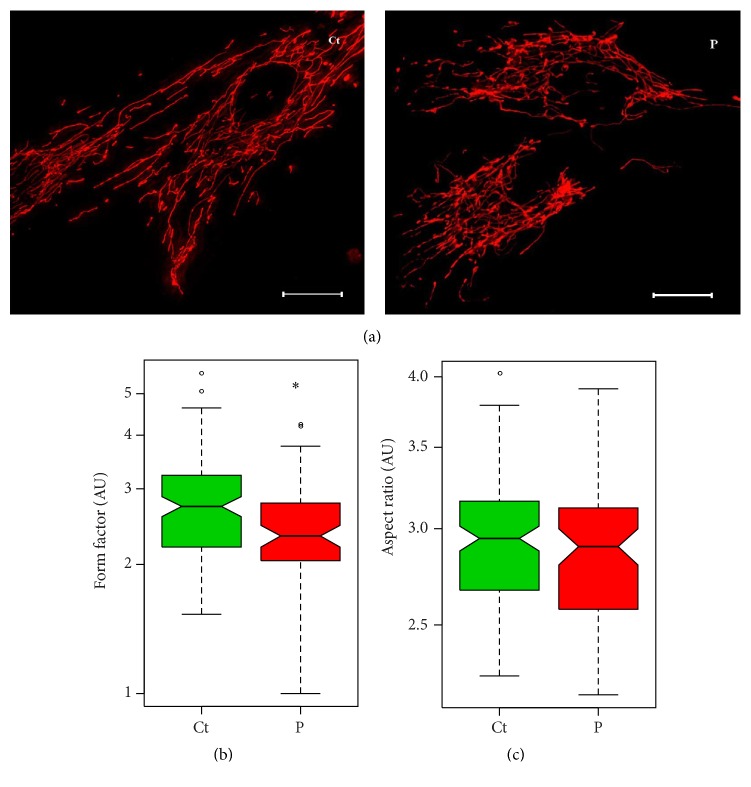
Mitochondrial network analysis of patient-derived and control fibroblasts. Mitotracker Red and live-cell microscopy were used to visualize the mitochondrial network. (a) Representative images of control fibroblasts (left) and patient fibroblasts (right). Scale bars = 20 *μ*m. All images were assessed in regard to the degree of mitochondrial branching (form factor) and degree of mitochondrial elongation (aspect ratio). The distribution of these parameters in grouped patient-derived (P) and grouped control (Ct) fibroblasts are represented on logarithmic scale in boxplots, *N* = 40. (b) Comparison of form factor, which was significantly lower in patient cells than in control cells (*p* = 0.0304). (c) Comparison of aspect ratio, which was similar in patient and control cells (*p* = 0.1638). ^*∗*^ *p* < 0.05; °, outlier; AU, arbitrary units; *N*; cells analyzed.

As parkin is involved in the regulation of mitochondrial dynamics, mitochondrial morphology was assessed in all* parkin*-mutant and control fibroblasts by means of live-cell microscopy and image analysis. Approximately 40 cells were analyzed from each fibroblast cell line, and representative images of* parkin*-mutant and control fibroblasts are shown in Figure 3(a). Each image was assessed with regard to form factor (degree of mitochondrial branching) and aspect ratio (degree of mitochondrial elongation). The form factor of patient-derived fibroblasts was significantly lower than that of control cells (*p* = 0.0304; Figure 3(b)), which is consistent with a more fragmented mitochondrial network. No significant differences were observed between the aspect ratios of patient-derived and control fibroblasts (*p* = 0.1638; Figure 3(c)).

### 3.3. Mitochondrial Membrane Potential (Δ*ψ*_*m*_) Was Similar in* Parkin*-Mutant and Control Fibroblasts

**Figure 4:**
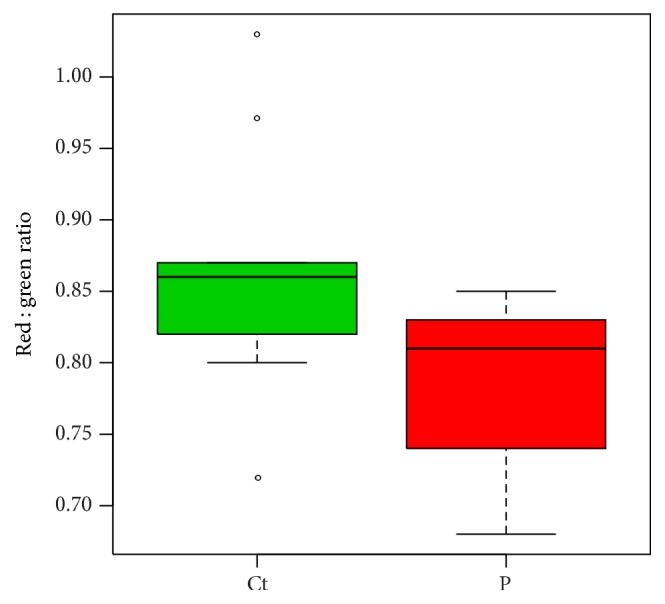
Relative Δ*ψ*_*m*_ of patient-derived and control fibroblasts. Relative Δ*ψ*_*m*_ was determined by JC-1 red : green fluorescent emission ratios for each fibroblast cell line in three experimental runs. Fibroblasts from P1 were not available; results pertain to a comparison of P2 and P3* parkin*-mutant fibroblast with the three controls. Similar Δ*ψ*_*m*_ was seen for patient-derived (P) and control (Ct) fibroblasts (*p* = 0.3285). °, outlier; Δ*ψ*_*m*_, mitochondrial membrane potential.

Given that Δ*ψ*_*m*_ is a central parameter of mitochondrial integrity, it was decided to assess Δ*ψ*_*m*_ in the fibroblast cell lines. The fibroblasts were stained with the JC-1 potentiometric dye, and the green and red fluorescent emissions of each cell population were simultaneously measured by means of flow cytometry. Differences in Δ*ψ*_*m*_ were detected by dissimilarities in red : green florescent emission ratios. The obtained red : green florescent emission ratios of the patient-derived and control fibroblasts are graphically illustrated in Figure 4. No significant differences in Δ*ψ*_*m*_ were observed for* parkin*-mutant and control fibroblasts (*p* = 0.1533).

### 3.4. *Parkin*-Mutant Fibroblasts Have Increased Cellular Growth While Being More Susceptible to Mitochondrial Insult

**Figure 5:**
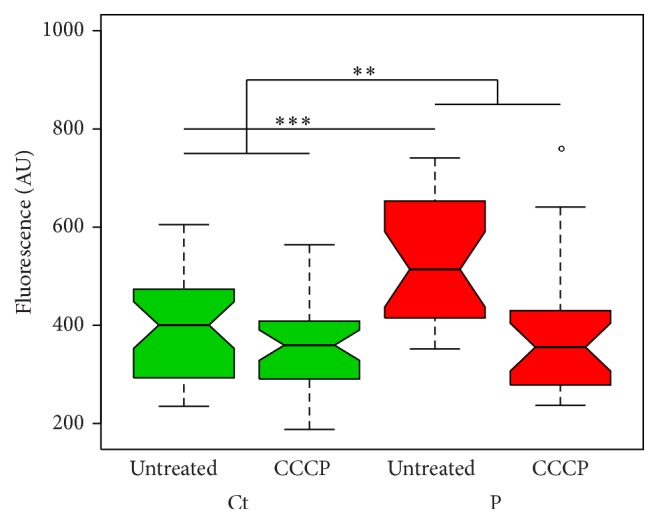
Cell growth in patient-derived and control fibroblasts under basal (untreated) and CCCP-stressed conditions, as assessed by a CyQUANT assay. Boxplots depict grouped patients (P) and control (Ct) measurements for three experimental runs. Patient cells demonstrated higher cell growth under basal conditions (*p* = 0.0001). A comparison of the magnitude of the effect of CCCP treatment within each fibroblast group (i.e., with and without cellular stress) demonstrated that the growth of patient-derived fibroblasts was significantly more suppressed by CCCP than the growth of control fibroblasts (*p* = 0.0013). Fibroblasts from P1 were not available; results pertain to a comparison of P2 and P3* parkin*-mutant fibroblast with the three controls. ^*∗∗*^ *p* < 0.01; ^*∗∗∗*^ *p* < 0.001; °, outlier; AU, arbitrary units.

CyQUANT assays of cell growth were performed to determine whether the overall state of cellular health differed between* parkin*-mutant and control fibroblasts. As illustrated in Figure 5, cell growth was significantly higher in patient-derived fibroblasts than controls under basal conditions (*p* = 0.0001). Fibroblast cell growth was also assessed under conditions of cellular stress, as any differences between patient-derived and control cells may not be readily apparent under basal conditions. Here, the fibroblasts were treated with CCCP to induce mitochondrial impairment and subsequent parkin recruitment to the damaged mitochondria. It was found that cell growth was similar between patient and control fibroblasts even after CCCP treatment (*p* = 0.0922). However, a comparison of the effect of CCCP treatment within each fibroblast group (i.e., with and without cellular stress) demonstrated that the growth of patient-derived fibroblasts was significantly more blunted by CCCP compared to the growth of control fibroblasts (*p* = 0.0013). This is indicative of a heightened sensitivity to CCCP of* parkin*-mutant fibroblasts in comparison to control fibroblasts.

## 4. Discussion

A substantial body of evidence supports parkin's involvement in mitochondrial function. However, many of these studies rely on artificially overexpressed or recombinantly tagged parkin, which may introduce experimental artifacts [39, 40]. It is therefore pivotal to investigate parkin-associated mitochondrial effects in appropriate cellular models where parkin is expressed at endogenous levels. Patient-derived dermal fibroblasts are particularly suitable for this, as their use creates an* ex vivo* model system with the defined* parkin* mutations and age-accumulated cellular damage of patients, while being minimally invasive to donor individuals [24]. The present study functionally compared* parkin*-mutant fibroblasts from South African PD patients with wild-type control fibroblasts using a variety of assays of mitochondrial health and function.

Surprisingly, it was found that the rate of mitochondrial respiration was increased in* parkin*-mutant fibroblasts in comparison to control fibroblasts. In particular, the patient cells demonstrated markedly increased basal respiration, elevated ATP-coupled respiration, and a higher maximal respiratory rate. This is indicative of increased electron flow through the respiratory chain, which is coupled to elevated oxidative phosphorylation. The unanticipated increment in mitochondrial respiration is in contrast to numerous studies which have reported decreased respiratory activity in fibroblasts from PD patients with* parkin* mutations. For example, Mortiboys et al. [25] described significant impairment of mitochondrial complex I activity in* parkin*-mutant fibroblasts, which was linked to a loss of Δ*ψ*_*m*_ and decreased cellular ATP content. Similarly, Pacelli et al. [27] reported that both the basal and maximal respiratory rate were significantly decreased in fibroblasts with* parkin* mutations. Further investigation of the specific respiratory complexes contributing to the decline in respiratory flux demonstrated that activity of complexes I, III, and IV was significantly reduced in patient fibroblasts [27].

It is interesting to compare these results to a recent report by Zanellati et al. [29], who used a similar experimental approach to the present study to investigate mitochondrial respiration in* parkin*-mutant fibroblasts from four PD patients. The authors likewise observed increased basal and maximal respiration in patient cells; however, despite this increment, they reported significantly lower ATP-coupled respiration in mutants compared to controls. These findings were associated with accordingly reduced cellular ATP levels and impairment of Δ*ψ*_*m*_, suggesting that* parkin*-mutant fibroblasts had uncoupled mitochondria. In contrast, the present study revealed a significantly increased ATP-coupled respiration in* parkin*-mutants, without any deficit in ATP-coupling efficiency or impairment of Δ*ψ*_*m*_. The paradoxically improved ATP-coupled respiration in the absence of parkin seen in the present study likely reflects a compensatory response in these* parkin*-mutant fibroblasts.

The present study found marked differences between the mitochondrial network morphology of patient and control fibroblasts. Whereas the degree of mitochondrial elongation (aspect ratio) was comparable between patient-derived and control fibroblasts, the amount of mitochondrial branching (form factor) was significantly decreased in patients. This decrease in form factor is consistent with increased fragmentation of the mitochondrial network [41]. We have previously reported that mitochondrial networks were unaffected in P1, P2, and P3 fibroblasts [28]. However, this follow-up study used a larger control sample size and was therefore more powered to detect differences in network parameters.

In contrast to the results obtained here, Mortiboys et al. [25] found that fibroblasts with* parkin* mutations had a marked increase in mitochondrial branching, as quantified by the form factor, suggestive of increased mitochondrial fusion in the absence of parkin. Two other studies indicated that* parkin*-mutant and wild-type control fibroblasts demonstrated comparable form factors under basal conditions [26, 42]. However, both studies found that treatment with mitochondrial stressors (paraquat and valinomycin, resp.) decreased the form factor and induced mitochondrial network fragmentation in* parkin*-mutant and control fibroblasts; these decreases were only statistically significant in the fibroblasts with* parkin* mutations. These findings are supported by the results of Pacelli et al. (2011), who observed a noticeably more fragmented mitochondrial network in* parkin*-mutant fibroblasts even under basal conditions; however, this difference was not quantified in terms of form factor. The results obtained here support these findings, but not the contrasting findings of Mortiboys et al. [25].

Moreover, in the present study it was found that cell growth was significantly higher in the* parkin*-mutant fibroblasts under basal conditions, which is in contrast to the published literature. For example, Mortiboys et al. [25] reported that cell growth was similar in fibroblasts from controls and patients with* parkin* mutations, whereas Pacelli et al. [27] reported that* parkin*-mutant fibroblasts displayed significantly lower growth than control fibroblasts. Both of these studies reported cell growth under basal, unstressed conditions. It is conceivable that the increased cell proliferation in the absence of parkin observed in the present study is a result of a metabolic shift in response to parkin deficiency, which is known to promote cell proliferation in various cancers with* parkin* mutations [43]. Furthermore, while significant efforts were made to only use fibroblast cell lines at low and comparable passage numbers, we cannot exclude the possibility that some of the cell lines may have undergone spontaneous transformation, which is known to affect cell growth rates.

It is speculated that the significant increases in basal, ATP-linked, and maximal respiratory rates of* parkin*-mutant fibroblasts, as well as elevated growth rates of these cells, may be due to a compensatory effect. In fact, several compensatory responses to parkin deficiency have been described in the literature. Pacelli et al. [27] reported that the defective ATP production in* parkin-*mutant fibroblasts was compensated by an upregulation of PGC1-*α* protein expression, suggestive of a compensatory increase in mitochondrial biogenesis [44]. However, the expressions of several PGC1-*α* target genes directly involved in mitochondrial biogenesis (including* NRF1*,* TFAM,* and* COX II*) were unchanged or even decreased in patient-derived fibroblasts. Pacelli et al. postulated that an unknown posttranslational modification of PGC1-*α* modulated its function in* parkin*-mutant fibroblasts, preventing a compensatory increase in mitochondrial biogenesis. It is interesting to speculate that the unique genetic backgrounds of the fibroblasts in the current study may allow for the PGC1-*α*-mediated increase in mitochondrial biogenesis. This deserves further study as it may, in part, explain the conflicting results obtained here and by Pacelli et al.

Other studies have also implied a compensatory increase in mitochondrial biogenesis in parkin deficient fibroblasts. Grünewald et al. [26] investigated citrate synthase activity as an index of total mitochondrial mass and found that such activity was significantly higher in* parkin*-mutant fibroblasts than wild-type controls. Indeed, Grünewald et al. did not observe any impairments of mitochondrial complexes I–IV under basal conditions. Hence, increased citrate synthase activity and elevated mitochondrial biogenesis in general may explain the milder phenotype of* parkin*-mutant fibroblasts observed by Grünewald et al. While markers of mitochondrial biogenesis were not assessed in the present study, increased biogenesis may underlie the compensatory increase in mitochondrial respiration seen here. It is noted that a possible compensatory elevation of mitochondrial biogenesis in the parkin-deficient fibroblasts would be paradoxical: parkin is involved in the promotion of mitochondrial biogenesis; hence, these processes are expected to be decreased in the absence of parkin [45]. However, future investigation of the exact nature and mechanism of the respiratory compensation observed in* parkin*-mutant fibroblasts may reveal a more complex and nuanced view of parkin and its interaction with mitochondria.

These compensatory responses are likely dependent on cell- and tissue-specific metabolic capacity and adaptations [46]. Hence, the observations made here on patient-derived fibroblasts should not be extrapolated to possible effects in a neuronal environment, as neurons may be more restricted in their compensatory repertoire than dermal fibroblasts. Furthermore, many of the described functional roles of parkin are cell-type specific which will result in different functional effects of parkin deficiency in fibroblasts and neurons [47]. Ideally, the observations made in this study should be verified in a neuronal model, such as induced pluripotent stem cell- (iPSC-) derived neurons with* parkin *mutations.

We recognize several limitations of this study. The findings are limited by the small sample size of three patient-derived fibroblasts cell lines, of which only two were available for Δ*ψ*_*m*_ and cell growth assays. Furthermore, two of the patients recruited for this study were siblings. The small sample size reflects the scarcity of* parkin*-mutant fibroblast models, which is also apparent in the small sample sizes (two to six) of previous studies on* parkin*-mutant fibroblasts [25–29]. It is recommended that the findings reported here be verified in a larger group of patients and controls. We also recommend stringent quality control measures when performing functional assays in fibroblasts, considering that cell growth, respiration, and oxidative stress can be greatly influenced by cellular senescence, spontaneous transformation, or undetected mycobacterial infections.

The functional effects of parkin deficiency observed in this exploratory study were assessed in fibroblasts cultured under basal, unstressed conditions. Given parkin's important role in the cellular stress response, mitochondrial impairments in Δ*ψ*_*m*_ in* parkin*-mutant fibroblasts may only be readily observable under highly oxidative conditions, where the cells are more reliant on mitochondria for ATP production. Future studies should aim to compare the results obtained here to fibroblasts cultured under more stressed or oxidative conditions, particularly in regard to the mitochondrial respiration and Δ*ψ*_*m*_ analyses.

In conclusion, our results do not support the findings of impairment of mitochondrial respiration in* parkin*-mutant fibroblasts, while concurring with previous reports of altered mitochondrial dynamics in these cells. These preliminary findings suggest a compensatory response in the patient fibroblasts used in this study. Future studies should aim at investigating the molecular mechanism of the mitochondrial compensation in the absence of parkin; proteomic analyses of* parkin*-mutant fibroblasts may be particularly suitable to identify dysregulated biological processes. Insights derived from these studies may have important implications for therapeutic strategies aimed at preserving mitochondrial function in PD patients.

## Figures and Tables

**Table 1:** Genotypic and demographic characteristics of the six dermal fibroblast donors used in this study.

Identifier	Lab ID	*Parkin* mutation	Gender	AAO (years)	AAR^*∗*^ (years)	PD duration (years)
PD patients						
P1	53.44	Deletion exon 3-4 hom	Female	27	39	12
P2	P2	Deletion exon 4 hom	Female	27	54	27
P3	P3	Deletion exon 4 hom	Female	27	52	25
Controls						
Ct1	WT2	n/a	Female	n/a	62	n/a
Ct2	WT3	n/a	Female	n/a	56	n/a
Ct3	WT4	n/a	Female	n/a	44	n/a

^*∗*^The age of the donor at the time of skin punch biopsy. AAO, age at onset; AAR, age at recruitment; hom, homozygous; n/a, not applicable; PD, Parkinson's disease.
